# Congenital Bladder and Urethral Agenesis: Two Case Reports and Management

**DOI:** 10.1155/2020/2782783

**Published:** 2020-09-24

**Authors:** Salahaddin Delshad, Hadith Rastad, Parham Mardi

**Affiliations:** ^1^Department of Pediatric Surgery, School of Medicine, Iran University of Medical Sciences, Tehran, Iran; ^2^Department of Pediatric Surgery, Maryam Hospital, Karaj, Iran; ^3^Non-Communicable Diseases Research Center, Alborz University of Medical Sciences, Karaj, Iran; ^4^Student Research Committee, Alborz University of Medical Sciences, Karaj, Iran

## Abstract

**Background:**

Agenesis of the bladder and urethra is a rare congenital anomaly, with a very few living cases reported in the literature so far. *Case Presentation*. We are reporting two female patients (3 and 6 years old) with bladder and urethral agenesis who presented with urinary incontinence. In both patients, magnetic resonant imaging (MRI) revealed a case of bladder and urethral agenesis with normal ureters draining into the vagina. Patients underwent a neobladder and conduit creation surgery. The neobladder was constructed from the whole cecum and a part of the ascending colon, followed by an anastomose of the ureters into the neobladder in a nonrefluxing fashion; the appendix was used simultaneously as a continent catheterizable conduit. The two patients attained urinary continence postoperatively.

**Conclusion:**

We reported two cases of bladder agenesis, and for the first time, we have performed neobladder creation surgery using the cecum and ascending colon. One-year follow-up did not reveal any complications.

## 1. Background

While urological anomalies are among the most common congenital defects [1], bladder agenesis is an extremely rare congenital anomaly, with approximately 74 cases described with only 25 cases of live birth reported in the literature written in English until 2020 [2]. The live-birth patients are often diagnosed in infancy or early childhood, presenting with other anomalies, urinary incontinence, and recurrent urinary tract infection (UTI) [3]. Here, we have reported two cases of bladder and urethral agenesis with the bilateral ectopic ureteral opening into the vagina.

## 2. Case Presentation

### 2.1. Case 1

A 3-year-old girl with a history of urinary incontinence, fecal incontinence, and imperforated anus who previously underwent colostomy was referred to our clinic for possible surgical correction. She is the only child in the family of nonconsanguineous parents and was born full-term with spontaneous vaginal delivery and normal birth weight. The mother's age was 25 years at the time of pregnancy.

**Figure 1:**
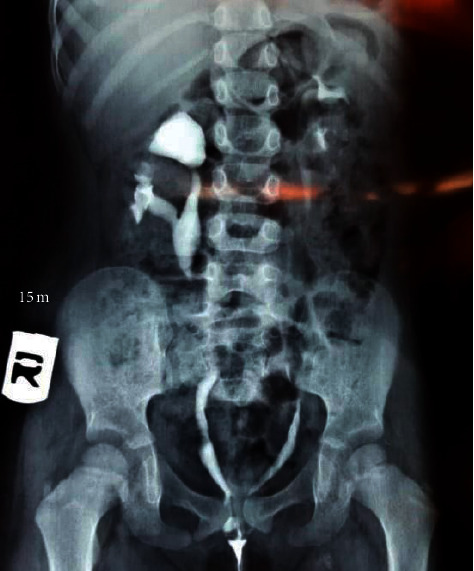
IVP with contrast showing agenesis of the bladder and ureters opening to the vagina.

Physical examination of the external genitalia demonstrated the normal appearance of the vulva, and ureteral orifices were not visible in the vagina, but we noted vaginal leakage of urine. Routine blood tests, including serum creatinine and urine tests, were normal. The absence of the bladder was noted on genitourinary ultrasonography. For further examination, IVP with contrast was performed, showing bilateral ectopic ureters opening directly into the vagina in the absence of the urinary bladder and urethra (Figure 1). MRI confirmed our diagnosis of bladder agenesis. It also identified a hypoplastic sacrum. Initially, the patient underwent a successful anorectoplasty after performing an EMG showing a normal functioning anal sphincter. One month later, colostomy was closed, and the patient achieved fecal continence despite having severe hypoplasia of the sacrum. After 14 months, when the patient was four years old, she underwent a successful neobladder and continent catheterizable stoma creation surgery. The stoma opens to the abdominal wall superior to the left anterior superior iliac spine. After surgery, the patient had both fecal and urinary continence.

### 2.2. Case 2

A 6-year-old girl with a history of urinary incontinence came to our clinic. The patient was fecal continent. She was born full-term with normal vaginal delivery of nonrelated parents and had two other healthy siblings. The external genitalia was normal on physical examination. At presentation, serum creatinine, blood urea nitrogen, and urea analysis tests were all normal. Bladder and urethral agenesis with bilateral ectopic ureters opening into the vagina was detected on IVP with contrast followed by abdominal and pelvic MRI. She had no other congenital anomalies. She underwent a successful creation of a neobladder when she was six years old, using the whole cecum, a part of the ascending colon, and a continent catheterizable stoma that opens to the abdominal wall superior to the anterior superior iliac spine. After surgery, the patient had both fecal and urinary continence.

### 2.3. Surgical Procedure

In both cases, we opted for a single-stage definitive method of procedure as explained below.

**Figure 2:**
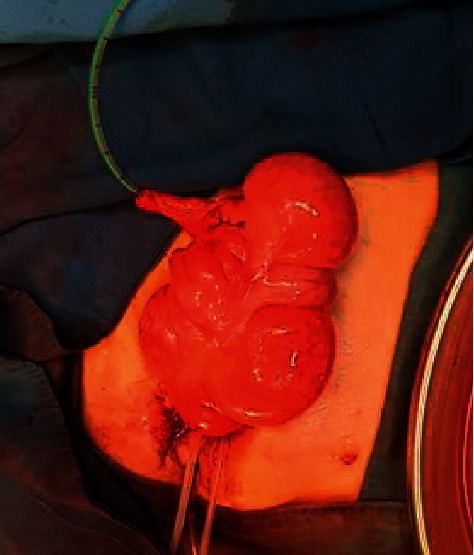
Normal saline passing through the conduit (appendix) after closing catheters of ureters.

**Figure 3:**
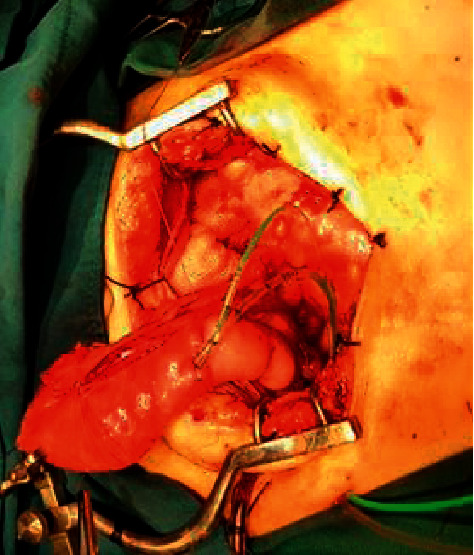
Neobladder creation surgery using the cecum and a part of the ascending colon.

Initially, the cecum and ascending colon were investigated using barium enema to detect any abnormalities. The volume of an absent vesicle was estimated at 300 mL. The reservoir will expand with time. The whole cecum and a part of the ascending colon were dissected off to make a urinary reservoir (neobladder), and the appendix was dissected with careful preservation of mesentery and blood vessels and used as a conduit. The tip of the appendix was anastomosed to the taeniae coli of the cecum by a flap-valve antireflux mechanism (Figure 2). The base was opened to the skin at RLQ; a catheter was passed into the conduit. Ureters were dissected from the vagina and attached by an antireflux mechanism (Figure 3). Then, two catheters were inserted into the ureters passing from the skin through an abdominal orifice 3 cm superior to the iliac crest. The neobladder was fixed posterior to the pubic bone. About 300 mL of normal saline was passed through the conduit after closing catheters of ureters to check for leakage.

At postoperative follow-up, both patients' urine analysis only showed RBC cast until day five after surgery. On day 7, ureter catheters were removed. Finally, contrast radiography did not detect any leakage or reflux, and patients were discharged on the eighth postoperative day, while the two patients attained urinary continence. Patients' parents were trained to drain the bladder using a catheter, at least eight times a day (intermittent self-catheterization). Patients were followed up for a year after surgery. In the following year after surgery, the patient was followed up by multiple contrast radiography and did not show any signs of stricture or prolapse. No other complications, such as UTI, were observed. The patients' parents did not give consent to scintigraphy.

## 3. Discussion

We presented two rare cases of bladder agenesis and ureteral opening into the vagina. Our cases both underwent successful neobladder creation.

Bladder agenesis is life noncompatible in the case of ureter obstruction. In live-birth cases like our cases, drainage of urine to the amniotic fluid through ectopic ureters preserves the renal function during the fetal period. [4] However, these patients will suffer from urinary incontinence after birth.

The etiology of bladder agenesis is unclear. In patients with bladder agenesis and hindgut abnormalities, as seen in our first case, the abnormal division of the cloaca is a more probable hypothesis rather than other raised hypotheses such as a primary developmental failure or secondary atrophy of the urogenital sinus [5]. Diagnosis of bladder agenesis is challenging. In order to diagnose bladder agenesis, it is essential to verify clinical findings and physical examinations with imaging studies. In this case, we have used both IVP with contrast and MRI. Although MRI is more informative for the diagnosis, IVP findings are necessary for the planning of the surgery [6].

The management of bladder agenesis is based on the severity and extent of the anomaly; previous literature has reported additional considerations and different adjunctive procedures in the management course due to a variety of concomitant anomalies [7].

Urinary reconstruction for bladder agenesis is accomplished by urinary diversions, such as ureterosigmoidostomy, stoma of ureters to the skin, or umbilicus [8]. The complications such as ureter necrosis, urinary incontinence, skin irritation, and ascites were frequent in these procedures. Although in this procedure the ileocecal valve is removed and hydroelectrolytic loss and malnutrition are expected, none of our patients shows gastrointestinal or malnutrition signs.

In both cases, we made a neobladder from the whole cecum and a part of the ascending colon, followed by anastomose of ureters into a neobladder using an antireflux mechanism. Then, we implanted the appendix as a catheterizable conduit into the urinary reservoir in a nonrefluxing mechanism. There is a controversy in the literature over the outcome of the antireflux mechanism. The antireflux mechanism showed a lower risk of pyelonephritis and bacteriuria [9, 10] but higher risk of the stone formation and stricture [11]. However, one randomized trial showed no difference between refluxing or antireflux anastomosis in terms of renal outcomes [12].

During the literature review, we found few other publications that addressed lower urinary tract reconstruction and attained continence in patients with bladder agenesis; Kasat et al. [3] managed a case of a five-year-old child with bladder agenesis and ectopic ureter opening into the vestibule using a continent ileocecal pouch (Penn pouch) with the Mitrofanoff principle [3].

More recently, Nazim and Zaidi [13] for an eight-year-old girl with a triad of complete agenesis of the bladder and urethra, solitary functioning left kidney, and an ectopic ureter opening into the vagina used both the sigmoid colon and ileum to made an orthotopic urinary reservoir, and the appendix was used as a catheterizable Mitrofanoff stoma [13].

Indiana pouch surgery is another option in these patients. In this procedure, the reservoir is created out of the ascending colon and the ileum. As ileum has a considerable amount of mucus secretion, electrolyte imbalances are expected in Indiana pouch surgery. Theoretically, using the ascending colon and cecum is associated with a lower risk of electrolyte imbalances [14].

The advantages of the urinary continent and antireflux mechanisms inherent to this procedure have been well documented. The use of the appendix as the continent catheterizable conduit is common in the treatment of patients who need lower urinary reconstruction due to its availability and independent blood supply [15].

## 4. Conclusion

Urinary bladder agenesis is a very rare congenital condition that is associated with multiple anomalies. It should be considered in patients with unexplained or atypical UTIs because early diagnosis and neobladder creation surgery can prevent or delay the progression to chronic kidney disease. We discussed two successful neobladder creation with the attainment of urinary continence in our patients with bladder agenesis.

## Data Availability

No data have been submitted to any open-access databases. All data supporting the study are presented in the manuscript or available upon request.

## Abbreviations

UTI: Urinary tract infection
IVP: Intravenous pyelogram
MRI: Magnetic resonance imaging
EMG: Electromyography
RLQ: Right lower quadrant (of the abdomen)
RBC: Red blood cells
CCC: Continent catheterizable conduit.
